# Dual Loading of Nanoparticles with Doxorubicin and Icotinib for the Synergistic Suppression of Non-Small Cell Lung Cancer

**DOI:** 10.7150/ijms.39172

**Published:** 2020-02-04

**Authors:** Ke Li, Wenhua Zhan, Min Jia, Yufeng Zhao, Yingguang Liu, Rajiv Kumar Jha, Liansuo Zhou

**Affiliations:** 1 Shaanxi Key Laboratory of Brain Disorders, Shaanxi Key Laboratory of Ischemic Cardiovascular Disease, Institute of Basic and Translational Medicine, Xi'an Medical University, Xi'an, 710021, Shaanxi, China; 2 Department of Radiotherapy, General Hospital of Ningxia Medical University, Yinchuan, 750004, Ningxia China; 3 Key Laboratory of Biomedical Information Engineering of Education Ministry, School of Life Science and Technology, Xi'an Jiaotong University, Xi'an 710049, Shaanxi, China; 4 Department of Basic Medical Science, Xi'an Medical University, Xi'an, 710021, Shaanxi, China; 5 College of Clinical Medicine, Xi'an Medical University, Xi'an, 710021, Shaanxi, China

**Keywords:** non-small cell lung cancer, combination chemotherapy, nanoparticles, icotinib, doxorubicin

## Abstract

**Background**: Combination chemotherapy plays an important role in the clinical therapy of non-small cell lung cancer (NSCLC). However, the pharmacokinetic differences between drugs are an insurmountable barrier in traditional treatment. For the synergistic therapy of NSCLC, synergistic nanoparticles (EDS NPs) loaded with both an EGFR inhibitor and doxorubicin (DOX) were designed and prepared.

**Methods**: Erlotinib, apatinib and icotinib were evaluated for optimal combination with DOX in treatment of NSCLC via CCK-8 assay. Then the cationic amphipathic starch (CSaSt) and hyaluronic acid (HA) were applied to coencapsulate DOX and EGFR inhibitor to form the EDS NPs. EDS NPs were evaluated in NSCLC cell lines (A549, NCI-H1975 and PC9) and NSCLC xenograft mouse models.

**Results**: Icotinib was found to be the optimal synergistic drug in combination with DOX in the tested. Subsequently, icotinib and DOX were coencapsulated in the NPs. EDS NPs were roughly spherical with an average size of 65.7±6.2 nm and possessed stable loading and releasing properties. In the *in vitro* investigation, EDS NPs could efficiently deliver payloads into cells, exhibited cytotoxicity and produced strong anti-migration properties. *In vivo* hypotoxicity was confirmed by acute toxicity and hemolytic assays. The *in vivo* distribution showed that EDS NPs could enhance accumulation in tumors and decrease nonspecific accumulation in normal organs. EDS NPs significantly promoted the *in vivo* synergistic effects of icotinib and DOX in the mouse model.

**Conclusions**: The study suggests that EDS NPs possess noteworthy potential for development as therapeutics for NSCLC clinical chemotherapy.

## Introduction

Lung cancer is a worldwide problem at present. According to cancer statistics for 2019, lung cancer accounts for approximately 13% of cancer incidence and 23% of mortality in the United States 1. NSCLC is the major type of lung cancer, accounting for approximately 80-85% of lung cancer cases 2. Most patients with NSCLC are diagnosed at an advanced stage, which reduces the opportunity for surgery. Although chemotherapy remains the primary standard treatment option for NSCLC in the clinic, severe systemic toxicity seriously affects the prognosis and quality of life of patients 3. Currently, epidermal growth factor receptor (EGFR) is one of the most important therapeutic targets of NSCLC. EGFR is overexpressed in more than half of NSCLC patients 4. EGFR is related to multiple mechanisms involved in tumor occurrence and development, including proliferation, migration, invasion and apoptosis 5. To date, various EGFR inhibitors have been developed as chemotherapeutic agents against NSCLC, and afatinib, gefitinib and erlotinib are the preferred first-line treatments in patients with advanced NSCLC 6. Despite satisfactory curative effects, drug resistance to EGFR inhibition inevitably occurs 7. Various mechanisms of resistance have been reported; however, the complete process is still not well understood 8. Hence, traditional single drug chemotherapy has not met the requirements for successful NSCLC treatment.

Combination chemotherapy could affect multiple pathways and mechanisms through different therapeutic agents, which have been applied in the clinical treatment of cancers and have been of benefit to cancer patients 9. Various studies have focused on combination chemotherapy of NSCLC. Lee and colleagues reported that erlotinib combined with cisplatin could efficiently induce apoptosis in erlotinib-resistant lung cancer 10. The Okabe group demonstrated that gefitinib and 5-fluorouracil have synergistic effects in some NSCLC cell lines 11. Kim et al. 12 found that a combination of lapatinib and cetuximab was able to produce synergistic effects in NSCLC cell lines. Many randomized and controlled trials of EGFR inhibitors combined with chemotherapeutic drugs have been conducted for the treatment of NSCLC; however, these combinations did not show definitely curative advantages compared with single drug treatments 13-15. Thus, drugs used in combination should act through different pathways and mechanisms to avoid disadvantages when used in combination and to effectively act in synergy while decreasing overlapping toxicity. To ensure optimal effectiveness, one drug should increase the sensitivity of cancer cells to other drugs in the combination 16. Lee et al. 17 demonstrated that an EGFR inhibitor (erlotinib) could effectively enhance the apoptotic response to DOX. The study suggested that the combination of DOX and EGFR inhibitors may encourage a synergistic effect. Nevertheless, two main barriers, the differences in the pharmacokinetic and the synergistic properties of drugs, were limiting the application and improvement of combination chemotherapy. The primary cause of this is the physicochemical differences of drugs, and these differences cannot be eliminated 18,19. Therefore, to meet this challenge, various novel technologies have been developed. The development of nanotechnology could solve the problems presented by the use of multiple therapeutic agents 20.

A nanocarrier could deliver multiple drugs at an appropriate ratio and dose, which would effectively enhance the synergistic effects of drugs while decreasing toxicity in normal organs and tissues 21. Numerous studies focused on nanoparticles in the treatment of NSCLC and attained obvious success. Chen et al. 22 used PEG-PLA as an encapsulation material to prepare NPs for erlotinib and fedratinib codelivery and evaluated the synergistic effects in fedratinib-resistant NSCLC mediated by the suppression of the JAK2/STAT3 pathway. Su and colleagues developed a nanocarrier system for gene and chemotherapy combination therapy of NSCLC cell lines 23. Chen et al. 22 prepared PEI-coated PLGA NPs that coencapsulated paclitaxel and siRNA, and these NPs suppressed Stat-3 expression and induced apoptosis in the NSCLC cell line A549 24. A novel dual drug-loaded nanoparticle was prepared for the treatment of K-RAS NSCLC, which has low sensitivity to current clinical therapies. The drugs used in the NPs were ganetespib and Pt (MCO)_2_ 25. Sulthana et al. 26 reported the use of a multifunctional nanocarrier loaded with DOX and ganetespib for the diagnosis and treatment of NSCLC. In a previous study, our group developed nanocarriers for the dual encapsulation of drugs for antitumor treatment. The NPs were coloaded DOX and apogossyplone and had an adjustable drug dose and ratio. Moreover, the outer material consisted of HA, which could provide a tumor target. In the study, *in vivo* tumor suppression was evaluated by using a PC-3 tumor-bearing mouse model. The NPs effectively enhanced the inhibition of tumor progression in mice and decreased side effects 27.

In the present study, three EGFR inhibitors (erlotinib, apatinib and icotinib) were evaluated to determine the optimal combination with DOX for the treatment of NSCLC cell lines (A549, NCI-H1975 and PC9). Among these, the combination of erlotinib and DOX has been reported to produce a synergistic effect in several breast cancer cell lines, including BT-20, m-453 and MCF-7 19. Apatinib was shown to overcome cancer multidrug resistance when combined with DOX 28. Additionally, apatinib exhibited a synergistic effect with DOX in soft tissue sarcomas 29. Icotinib combined with chemotherapeutic agents in patients with NSCLC could improve progression-free survival and overall survival 30. Subsequently, the CSaSt and HA were used for the dual coencapsulation of drugs through self-assembly to construct EDS NPs. When the EDS NPs were prepared and characterized, three human NSCLC cell lines were used for the evaluation of *in vitro* cell suppression and internalization, and BALB/c mice and NSCLC xenograft mouse models were used for evaluation of *in vivo* toxicity, delivery and antitumor activity. The study demonstrated the improved synergistic effects of EGFR inhibitors and DOX in NSCLC treatment and the excellent prospects for the use of EDS NPs for the clinical chemotherapy of NSCLC.

## Materials and Methods

### Materials

DOX, erlotinib, apatinib and icotinib were purchased from Sigma Int (MO, USA). The CCK-8 kit, Protein Extraction kit, BCA kit, TUNEL Apoptosis Detection kit, and Cell Apoptosis Detection kit were purchased from Beyotime Biotech Corp. (Shanghai, China). The DAPI kit was purchased from Bioworld Inc (MN, USA). The primary antibodies (Bcl-2, Bax, Caspase 3, Caspase 9, β-actin) and horseradish peroxidase-conjugated goat anti-mouse IgG were ordered from Cell Signaling Co., Ltd. (MA, USA). High-glucose DMEM, trypsin (0.25%) and antibiotics were obtained from HyClone Co. (UT, USA). FBS was purchased from Tianhang Co., Ltd. (Hangzhou, China). Experimental consumables, such as cell culture dishes, well plates and pipettes, were purchased from Corning Int. (NY, USA). HA (6.2 kDa) was purchased from Dongfang Chemical Corp. (Zhenjiang, China). CSaSt was prepared by our lab. Coumarin-6, Triton X-100, IR-780, Coomassie brilliant blue and crystal violet were purchased from Aladdin Corp. (Shanghai, China). Other reagents and labware were provided by Dingsheng Co. (Xi'an, China).

The human NSCLC cell lines A549, NCI-H1975, PC9, and human umbilical vein endothelial cells (HUVECs), and human lung fibroblast cell line (IMR-90) were provided by ATCC. The BALB/c mice and BALB/c-nu/nu mice were obtained from Charles River Labs (Beijing, China).

### Evaluation of the synergistic effects of different drug combinations

To obtain optimal synergistic anti-NSCLC effects, erlotinib, apatinib and icotinib were combined with DOX to treat NSCLC cells. Three NSCLC cell lines were used in this study, including A549, NCI-H1975 and PC9. The medium used for cell culture was complete high-glucose DMEM (containing 10% FBS and 1% antibiotics). All cells were incubated in an incubator (3111, Thermo Fisher, Shanghai, China) in 5% CO_2_ at 37°C. A CCK-8 assay was used to evaluate the inhibition of proliferation. Initially, the NSCLC cells were treated with DOX, erlotinib, apatinib and icotinib, and the IC_50_ values were calculated. Then, different proportions and concentrations of the therapeutics in combination were used for the investigation of the synergistic effects. The dose reduction and combination index (CI) reflected the degree of synergy 31.

### Preparation and performance of EDS NPs

The process of EDS NPs construction was described in our previous study 27. EDS NPs were assembled by using DOX NPs and EGFR inhibitor (icotinib) MCs via electrostatic absorption. The outer component, consisting of the DOX NPs, was fabricated from DOX and HA via electrostatic interactions. DOX solution was added dropwise into HA solution for the construction of the DOX NPs. The amount of HA added should be more than 10 times the amount of DOX to ensure that the assembly is complete. Subsequently, icotinib micelles were constructed via hydrophobic assembly. The EGFR inhibitor and CSaSt were dissolved in DMSO at a ratio of 1:4. Then, the mix solution was added dropwise into water and stirred for 10 min. The icotinib MC solution was added to the DOX NPs while stirring. After 20 min of stirring, the EDS NP solution was dialyzed to remove DMSO and other impurities. The ratio of DOX NPs to icotinib MCs was determined by measuring the optimal synergistic proportion of DOX and EGFR inhibitor.

### Characteristics and roperties of EDS NPs

The hydrodynamic size, zeta potential and polymer dispersion index (PDI) of the EDS NPs were measured by laser light scattering (DLS). The morphology and dried sizes of the EDS NPs were observed by transmission electron microscopy (TEM) (JEM-2100F, JEOL, Japan). The amounts of DOX and icotinib were measured by fluorospectrophotometry and HPLC, respectively. The encapsulation efficiency (EE) and drug loading (DL) were calculated.

### Investigation of release and stability* in vitro*

The* in vitro* release of EDS NPs was evaluated by dialysis. DOX is hydrosoluble, which could directly reflect the release process *in vitro*. Free DOX solution was used as the control. HAase was added into the EDS NPs to evaluate their release during HA decomposition. However, the icotinib is water insoluble. Its *in vitro* release is too difficult to evaluate via aqueous dialysis. Hence, the release of icotinib was indirectly detected after dialysis. The stability of the EDS NPs was measured according to size changes under different conditions, including exposure to PBS, medium and FBS. The aim of the test was to ensure that the EDS NPs could be used in the subsequent *in vitro* and *in vivo* experiments.

### *In vitro* suppression of proliferation

The* in vitro* suppression effect of the EDS NPs was evaluated by CCK-8 and clone formation assays. In the CCK-8 assay, EDS NPs and equal concentrations of both drugs were used to treat three NSCLC cell lines (A549, NCI-H1975 and PC9), respectively, and then the inhibition effects were compared. As the control, the human normal cell lines, HUVECs and IMR90, were treated with same conditions. In the clone formation assay, A549 cells were employed for the experiment. A total of 500 A549 cells were cultured in each dish. The dishes were treated with EDS NPs, DOX, icotinib and both drugs in combination. The concentrations of the drugs in the different treatment groups were equal. All dishes were incubated at 37 ºC in 5% CO_2_ for 72 h. Then, the stale medium was replaced by fresh medium with 20% FBS. After 5 d, the cells were fixed and then stained with crystal violet. The clones in each group were quantified.

### *In vitro* inhibition of migration

A transwell assay was utilized to evaluate cell migration. The A549 cells were treated by EDS NPs, DOX, icotinib and dual drugs, respectively. Then, a total of 200 μL of treated A549 cell suspension (1×10^5^ cells/mL) was seeded into the upper chamber of each Millicell hanging cell culture insert (8.0 μm, Millipore, Darmstadt, Germany). The cell suspension was made in medium without FBS. Subsequently, the inserts were placed into wells containing medium with 40% FBS. The cells were incubated at 37 ºC in 5% CO_2_ for 24 h. The cells in the inserts were fixed with methanol and stained with crystal violet. Then, the cells in the upper chamber were wiped away. Finally, the cells in the membranes of the inserts were observed by microscopy.

### Cell internalization and affinity evaluation

The fluorescence-labelled EDS NPs were used for the evaluation of cell internalization and affinity. The hydrophobic fluorescent dye coumarin-6 was replaced with icotinib for the labelling of the NPs. A549 cells were seeded into confocal cell dishes. When the cells grew to 50% confluence, coumarin-6-labeled NPs were added into the dishes. At different time points, the dishes were treated with 4% paraformaldehyde solution, and the cell nuclei were stained using a DAPI kit. The cells were observed by confocal microscopy (TCS SP5 II, Leica, Germany). HA was utilized for affinity evaluation via the competitive blocking of endocytosis. Initially, A549 cells were coincubated with 2% HA for 2 h, after which the NPs were added. After incubation, the fluorescence intensity was compared with that in cells without blocking. The fluorescence intensities were measured and quantified by using ImageJ software.

### *In vivo* toxicity of EDS NPs

Acute toxicity and hemolysis assays were used to evaluate *in vivo* toxicity. For the acute toxicity assay, there were 2 groups of mice, and 5 male and 5 female BALB/c mice were in each group. The mice were fed for 3 d. Then, the mice in the 2 groups were intravenously injected with either a combination of both drugs or the EDS NPs. The NPs and drugs in free form were administered at the same dosage: DOX at 5 mg/kg and icotinib at 50 mg/kg. The mortality within 2 w was recorded. The surviving mice were euthanized. The organs, which were collected from the surviving mice, were removed for histopathological analysis. For the hemolysis assay, whole blood was extracted from BALB/c mice and immediately treated with heparin. Then, the blood was equally divided into 6 tubes and treated with Triton X-100 (1%, v/v), both drugs in combination (DOX: 20 mg/ml, DOC: 0.05 mg/ml), EDS NPs (at the same concentration used for the drug combination), DMSO (at the same concentration used for the drug combination), empty NPs or saline, respectively. The samples were placed in a 37°C water bath for 2 h. Subsequently, the samples subject to centrifugation (13000 rpm, 15 min), and then the supernatants were collected and evaluated at 394 nm 32. All animal experiments were conducted according to the Guidelines for the Use and Care of Experimental Animals at Xi'an Medical University, which reference internationally accepted laboratory animal use and care guidelines (2011). The study process was supervised by the Laboratory Animal Administration Committee of Xi'an Medical University (No. XYACU2017-213).

### NSCLC xenograft mouse model

Male BALB/c-nu/nu mice (4 w of age) were fed in a SPF animal room. A total of 100 μL of A549 cell suspension, at a concentration of 5×10^6^/mL, was subcutaneously injected into the flank of each mouse. After 1 week, the tumors were measured, and when the tumors reached an adequate size, the model animals were used for further *in vivo* investigations.

### *In vivo* target delivery

Optical *in vivo* imaging was performed. To observe the *in vivo* distribution of the EDS NPs, the NIF fluorescent dye IR-780 was used to label the NPs. Two NSCLC xenografted mice were selected to evaluate the *in vivo* delivery of the EDS NPs. One mouse was injected with 200 μL of IR-780-labeled EDS NPs via the caudal vein. The other was injected with 200 μL of IR-780 solution at an equal dosage. Then, the mice were observed by an IVIS imaging system (Perkin Elmer, Waltham, MA, USA). The *in vivo* distribution of the NIF signal was captured and counted at several time points. When the observations were finished, the mice were euthanized, and the organs were harvested for the evaluation of NIF signal in tissues. In order to overcome the individual difference of mice, and further demonstrate the tumor targeting of EDS NPs, other two NSCLC xenograft mice were used for this experiment again. The dose of IR-780 in this case was higher than it in first experiment, and other details were followed by the method as described previously.

### Evaluation of *in vivo* tumor suppression

Thirty NSCLC mouse models were selected for the evaluation of the *in vivo* anti-tumor effects. When the average volume of the tumors reached approximately 200 mm^3^, the treatment was started. Initially, the mice were randomly divided into 5 groups. The first group was injected with saline as a control; the second group was injected with free DOX at a dosage of 2 mg/kg; the third group was injected with free icotinib at a dosage of 20 mg/kg; the fourth group was treated with a dual drug combination at dosages of 2 mg/kg and 20 mg/kg for DOX and icotinib, respectively; the last group was treated with EDS NPs at the same dosage used for the dual drug combination. The injection interval was 2 d, and the injections were carried out over 1 month. The tumor volumes and body weights were recorded continuously. Then, the mice were euthanized and the tumor tissues were removed for pathological investigation.

### Statistical analysis

All of the data are presented as the mean ± SD. The data were analyzed in GraphPad Prism 5.0 software. A P-value ≤ 0.05 indicated that there was a significant difference between the compared data.

## Results and Discussion

### Synergistic effects of erlotinib, apatinib and icotinib combined with DOX in NSCLC cell lines

As first-line chemotherapeutic agents for NSCLC, EGFR inhibitors play an important role. However, drug resistance and severe systemic toxicity seriously affect the prognosis and quality of life of patients 3,7. To overcome the defects of these drugs, various studies of combination chemotherapy for the treatment of NSCLC have been conducted 11,12,33. Previous research has indicated that EGFR inhibitors could enhance the curative effects of DOX in tumor cells 19 and indicated that the combination of DOX with an EGFR inhibitor was a potential synergistic chemotherapy for NSCLC. Hence, in the present study, various EGFR inhibitors, including erlotinib, apatinib and icotinib, were evaluated for their synergistic effects with DOX for the treatment of several NSCLC cell lines (A549, NCI-H1975 and PC9).

The results showed that the cytotoxicities of the different drugs were very high in the three cell lines. In general, DOX exhibited more effective inhibition than the EGFR inhibitors. A549 cells were more insensitive to apatinib, NCI-H1975 cells were sensitive to erlotinib and apatinib, and PC9 cells exhibited particular sensitivity to erlotinib and icotinib. When DOX was combined with the three EGFR inhibitors, the cytotoxicities were generally enhanced. The CI values reflected the significant differences between the combinations. The combination of DOX and erlotinib exhibited relatively similar synergistic effects in the three cell lines (Table 2). The addition of apatinib to DOX improved synergistic inhibition only in the NCI-H1975 cell line (Table 3). Icotinib combined with DOX had a greater synergistic effect than DOX combined with erlotinib, except in the PC9 cell line (Table 1). These results were observed in a 1:10 ratio of DOX and EGFR inhibitor, since, as referenced in a previous work, the use of both drugs at this ratio was more efficient for their encapsulation in the NPs. The combination of DOX and icotinib was used for further experiments.

### Detection of the characteristics and capabilities of the EDS NPs

**Figure 1:**
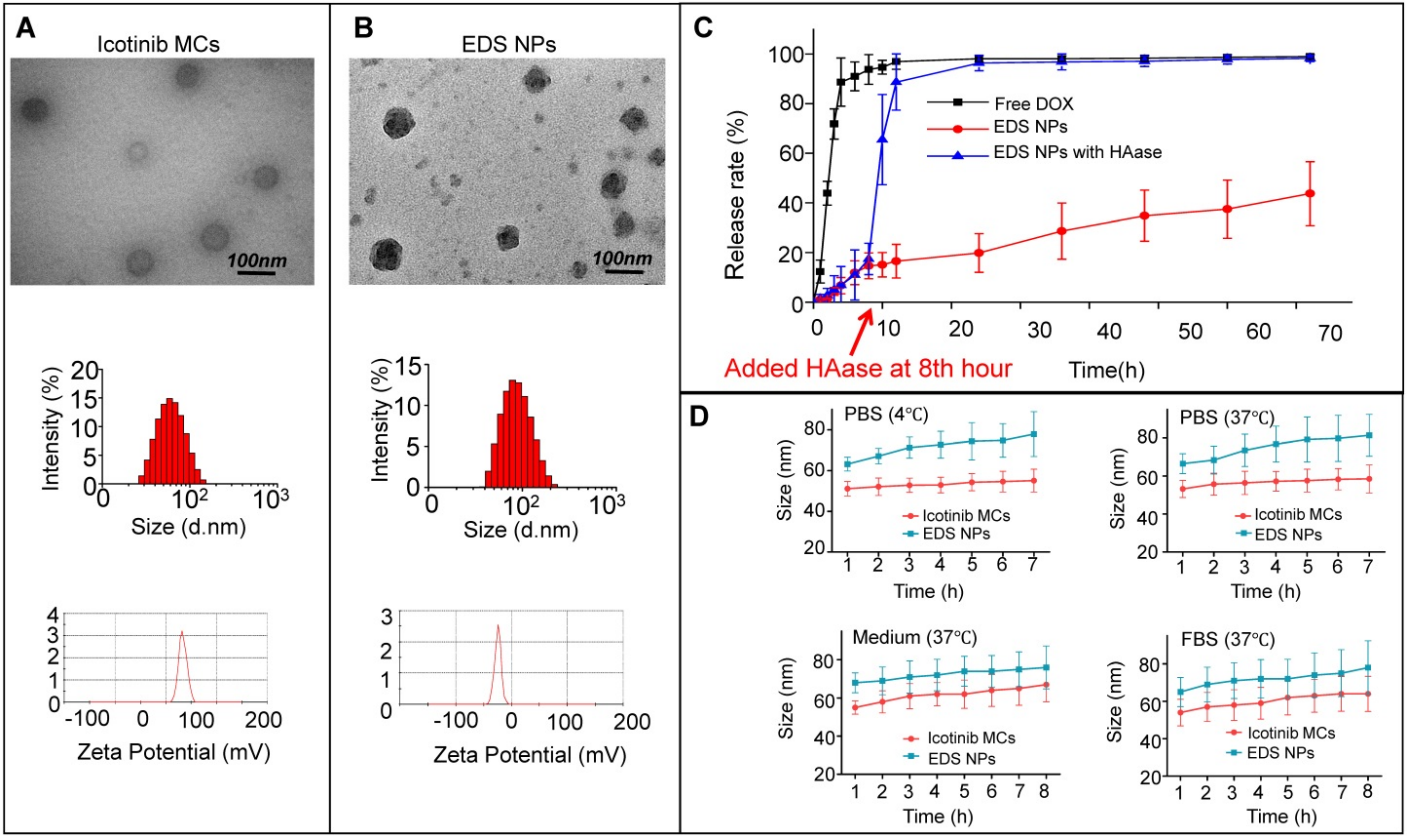
Characteristics of EDS NPs. TEM photos, size and zeta potential of Icotinib MCs (A) and EDS NPs (B). *In vitro* release of DOX in EDS NPs (C). *In vitro* stabilities of EDS NPs in PBS, medium and FBS (D).

To achieve the maximal synergistic effects, two obstacles needed to be addressed: the determination of the appropriate ratio and the concentration needed for treatment 21. The NPs used for drug delivery represented an advantageous method to overcome the barriers for combination chemotherapy. Various delivery systems based on NPs have been developed for cocktail chemotherapy 9. In the treatment of NSCLC, the NPs have also been widely used for the codelivery of chemotherapeutic agents, nucleic acids and antibody-based drugs 22,23,25,34. In our previous work, multifunctional NPs were designed for the synergistic treatment of tumors. NPs with an adjustable drug dosage and ratio were coloaded DOX and apogossyplone. In addition, synergistic cytotoxicity was demonstrated in A549 cells *in vitro*. The NPs effectively enhanced the inhibition of tumor progression in mice and decreased side effects 27. According to previous works, NPs for DOX and icotinib codelivery were designed and prepared. DOX and icotinib were encapsulated by HA and CSaSt, respectively, via a self-assembly process. The DOX-loaded NPs were absorbed onto the surfaces of icotinib MCs through electrostatic interactions. The sizes of the DOX NPs, icotinib MCs and EDS NPs were 11.2±6.5 nm, 53.8±4.1 nm and 65.7±6.2 nm, respectively. The polymer dispersion index (PDI) values for the three types of NPs were less than 0.25, and the PDI for the icotinib MCs was only 0.12. Moreover, the zeta potentials of the DOX NPs, icotinib MCs and EDS NPs were -22.9±1.6 mV, 33.1±2.4 mV and -22.3±3.7 mV, respectively. The differences in the zeta potentials further demonstrated that the assembly process was successful. As shown in Figure 1B, the TEM observations indicated that the EDS NPs were spherical particles with rough surfaces and possessed good monodispersity. The size of the NPs measured by dynamic light scattering was apparently larger than that determined based on the TEM photos because the hydration layer had evaporated during the preparation process prior to TEM observation. The diameter provided the EDS NPs the capacity of target delivery into solid tumors via the enhanced permeability and retention (EPR) effect, and the surface properties endowed the NPs with relative stability in blood 35,36.

The EE and DL of DOX were detected by fluorescence spectrophotometry, and the results were 90.1±2.9% and 1.3±0.3%, respectively. Via HPLC, the EE of icotinib was measured as 97.2±1.8%, and the DL was approximately 12%. The proportions of DOX and icotinib in the EDS NPs were consistent with the data regarding their synergistic effects. The *in vitro* release of drugs was evaluated by dialysis for DOX. As shown in the release curves (Figure 1C), EDS NPs exhibited sustained release during the experiment. Compared with free DOX, only 40% of DOX was released from the EDS NPs; however, the addition of HAase greatly accelerated its release. Icotinib is hydrophobic. After dialysis, the residual amount remaining accounted for 90% of the total. The stability of the NPs was reflected by their changes in size. As shown in Figure 1D, the EDS NPs remained stable in PBS, complete medium and serum during refrigeration and at physiological temperature. The results indicated that the EDS NPs exhibited excellent encapsulation capacity and stability; moreover, the NPs could tolerate enzymatic release. These properties supported the use of EDS NPs for subsequent *in vitro* and *in vivo* experiments.

### *In vitro* investigation of the suppression of proliferation, migration and endocytosis in NSCLC cells

**Figure 2:**
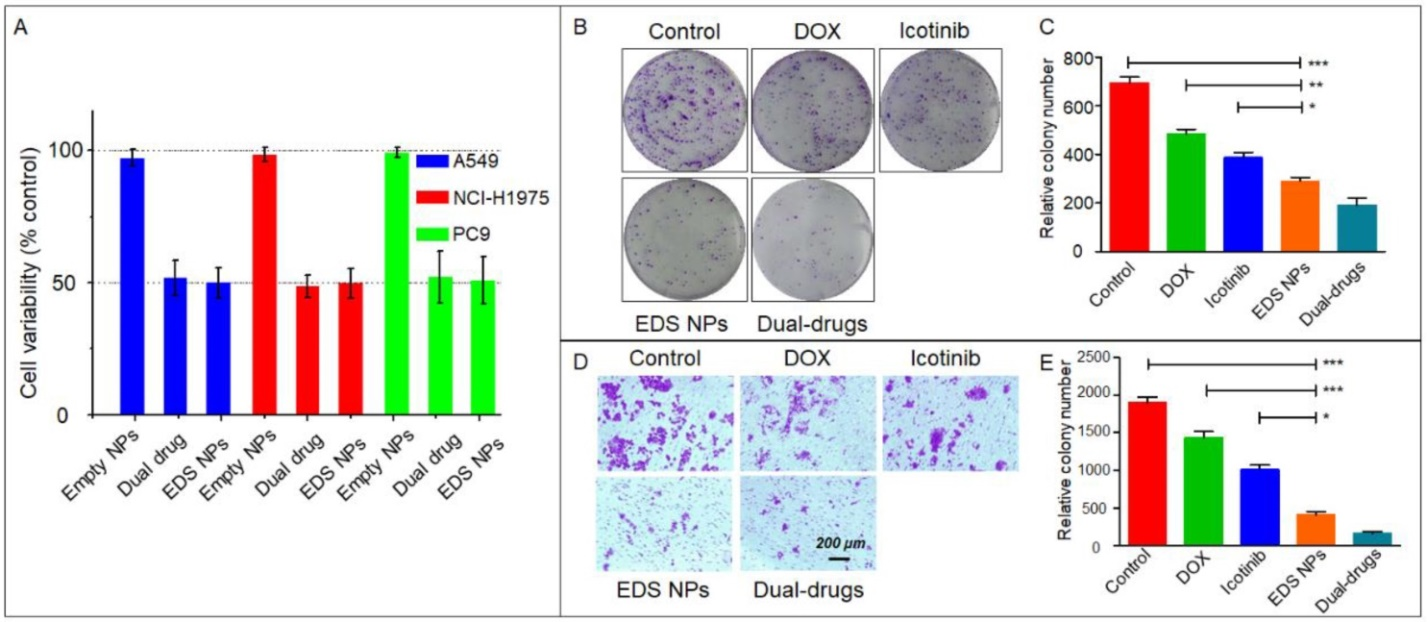
***In vitro* inhibition of EDS NPs.** Cytotoxicity of EDS NPs in three NSCLC cell lines (A). The results of clone formation assay of EDS NPs (B). The results of transwell assay of EDS NPs (C). Error bars represent the SD of the mean (n=3). The * indicated p < 0.05 in t-test, ** indicated p < 0.01, *** indicated p < 0.001.

The nanocarriers offered an option for combination chemotherapy that could overcome the differences in the pharmacokinetics of the drugs and achieve the maximal synergistic effect. Sulthana et al. 26 designed NPs for DOX and ganetespib codelivery for the diagnosis and treatment of NSCLC. Feng et al. 37 used time-staggered gold NPs for the coloading of erlotinib and DOX for synergistic chemophotothermal therapy. In addition, controlled-release NPs were used for the codelivery of apatinib and DOX 38. In the present study, the *in vitro* suppression of proliferation and migration were evaluated by CCK-8, clone formation and Transwell assays to verify the synergistic effect in NSCLC cell lines. The results indicated that EDS NPs effectively retained the synergistic effects of DOX and icotinib. As shown in Figure 2A, compared to combinations of the free drugs at equal concentrations, the EDS NPs produced the same inhibitory effects in all three NSCLC cell lines. Moreover, the *in vitro* cytotoxicity of EDS NPs was evaluated in two of normal cell lines, IMR90 and HUVECs. The results showed in Figure S1. The inhibitory effects of EDS NPs and dual drugs did not show significant difference in these normal cell lines. The results indicated that EDS NPs has cytotoxicity in normal cells. However, the *in vitro* result did not suggest that EDS NPs has side effect *in vivo*. Because, in cell culture condition, the EDS NPs was added in to medium directly. It could enter cell gradually, and released the payloads in the cytoplasm. Furthermore, the negative tumorous target, which based on EPR effect, could not implement. Hence, it did not indicate serious toxicity against normal tissues. The actual side effect will be evaluated in animal models. The clone formation assay further demonstrated that EDS NPs can effectively suppress proliferation in the A549 cell line. The number of clones in the EDS NPs-treated group was obviously greater than that in the single drug-treated and saline-treated groups, and there was no significant difference between the two drug combinations (Figure 2B and C). The anti-migration assay results are shown in Figure 2D and E. The number of transmembrane cells in the EDS NP-treated group and the dual drug combination group were significantly decreased compared with those in the single drug-treated groups. The EDS NPs with DOX and icotinib clearly exhibited synergistic effects.

**Figure 3:**
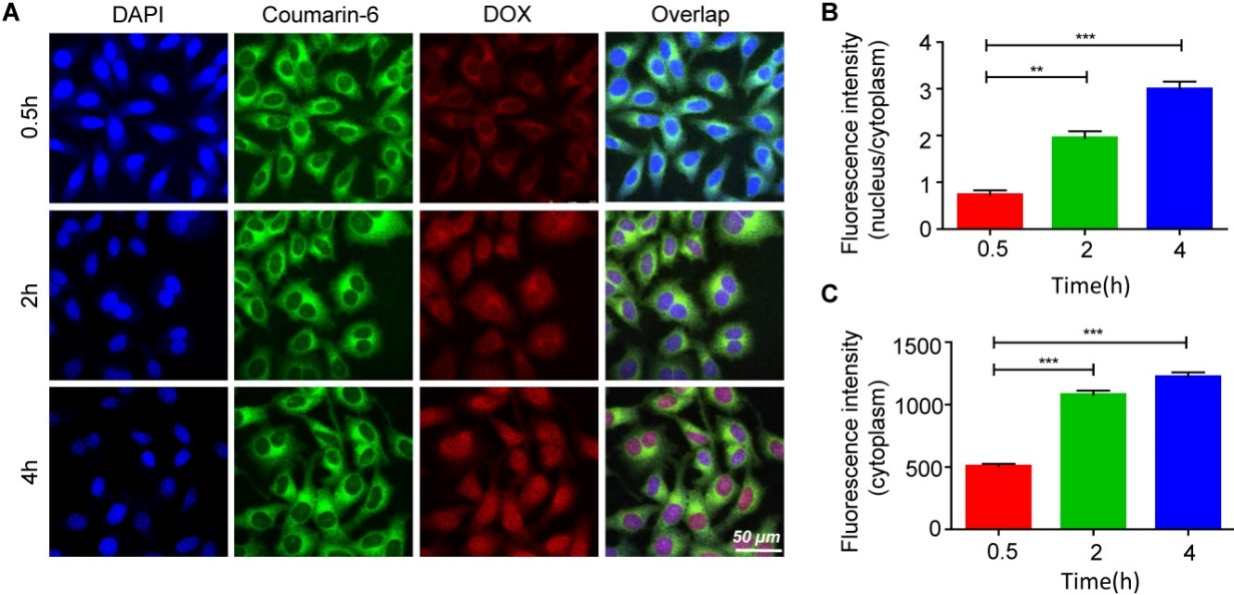
** Internalization and intracellular release of EDS NPs.** Confocal imaging of the fluorescent labelled EDS NPs (A). Quantitative analysis of fluorescent intensity in cell (B and C). Error bars represent the SD of the mean (n=3). The ** indicated p < 0.01 in t-test, *** indicated p < 0.001.

HA is widely used in drug delivery systems because it has ligand-receptor interactions with CD44 39. CD44 is expressed at low levels in normal tissues; however, it is pathologically highly expressed on the surfaces of tumor cells 40-42. Thus, the targeted delivery of the EDS NPs was performed by using HA. Figure 3A shows the process of internalization. The green fluorescence was derived from coumarin-6. The dye mainly stains the cell membrane and primarily accumulates in the cytoplasm. DOX emits red fluorescence, and it mainly binds to the nucleus. The changes in the distribution and intensity of the fluorescence signal could reflect the processes of internalization and intracellular release. In the first 30 min, both green and red fluorescence were concentrated in the cytoplasm, and the intensities were relatively weak. Then, as time went on, the intensity of fluorescence was increased, and the green and red fluorescence separated. After 2 h, DOX started to accumulate in the nucleus. After the 4th hour, the red fluorescence had equally accumulated in the cytoplasm and nucleus, and coumarin-6 remained in the cytoplasm. The results indicated that the NPs could effectively deliver the payloads into the cells, which were fully released.

**Figure 4:**
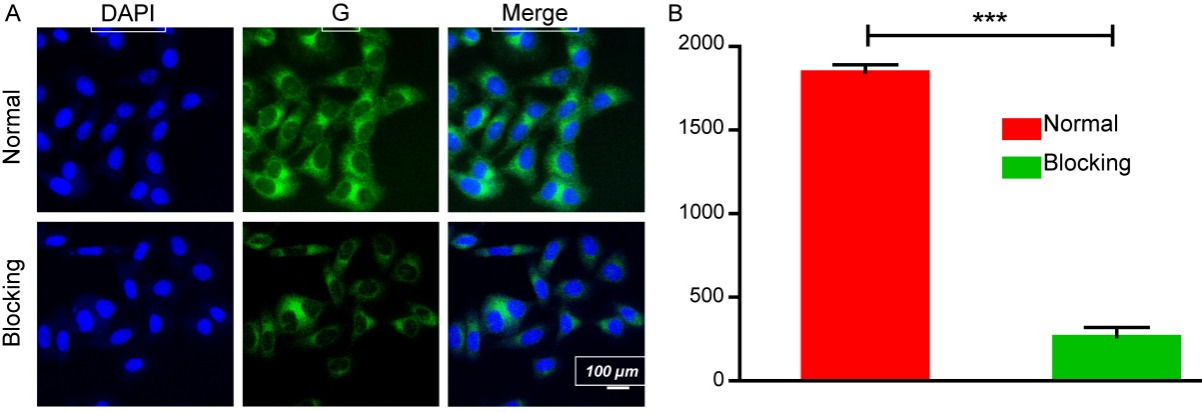
** Affinity of EDS NPs in NSCLC cell.** Confocal imaging of EDS NPs with/without HA blocking (A). Quantitative analysis of fluorescent intensity in cell (B). Error bars represent the SD of the mean (n=3). The *** indicated p < 0.001 in t-test.

The cell-targeted delivery of EDS NPs depended on ligand-receptor mediation via HA and CD44. A HA blocking test was used to investigate mediated endocytosis. As shown in Figure 4, the fluorescence intensity in the HA pro-treated group was significantly lower than that in the control group, suggesting that HA competitively suppressed the endocytosis of EDS NPs in the A549 cells. The results indicated that EDS NPs are capable of tumor target delivery, which was investigated in further experiments.

### *In vivo* targeted delivery

**Figure 5:**
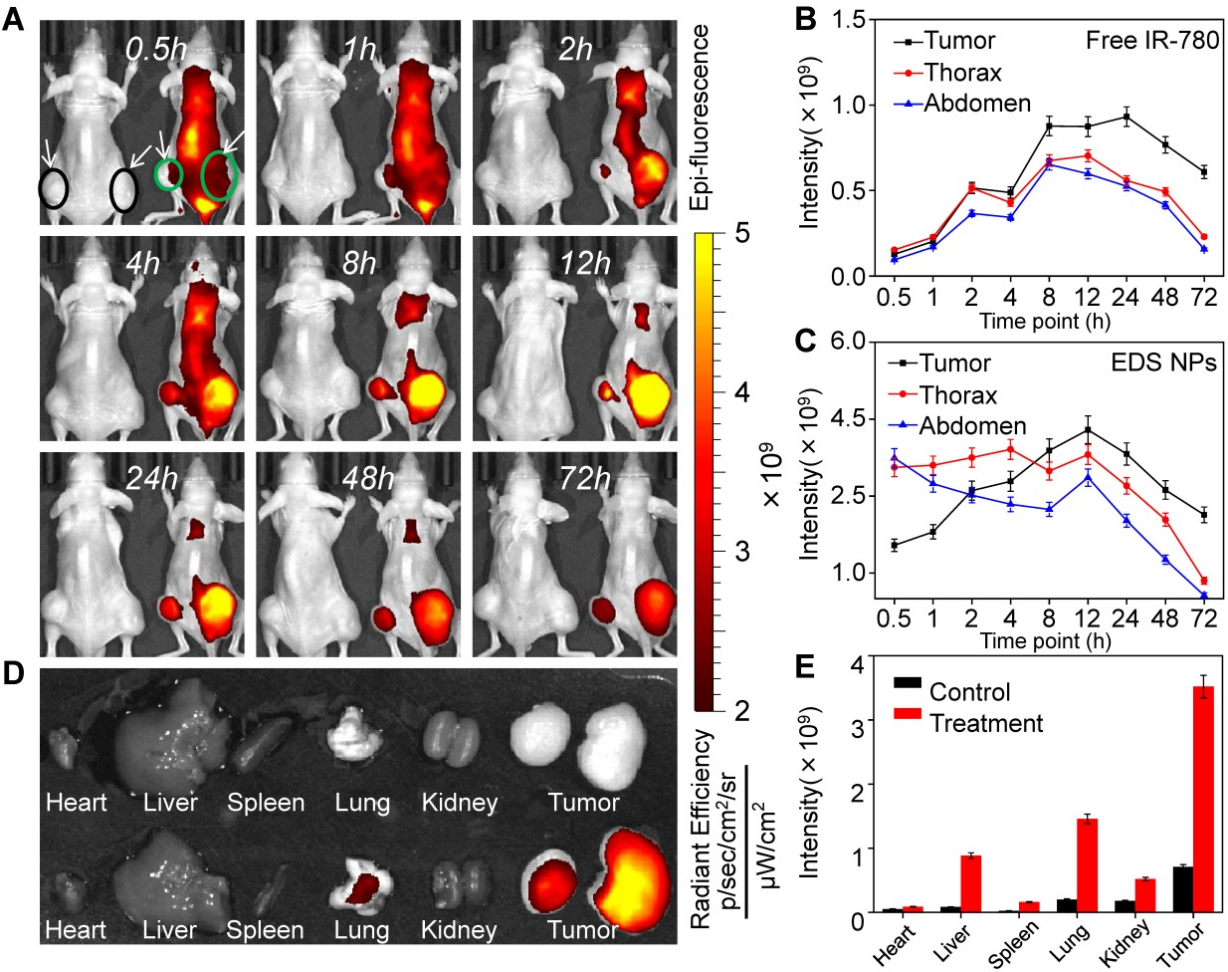
***In vivo* targeted delivery of EDS NPs.** The fluorescent signal distribution of EDS NPs during the experiment (A). Quantitative analysis of fluorescent intensity in mice (B and C). The fluorescent intensity in organs and tumors (D). Quantitative analysis of fluorescent intensity in tissues (E).

The primary function of NPs is the delivery of therapeutic agents into tumor tissues, which reduces their accumulation in normal organs and tissues 43. The *in vivo* distribution of the NPs was affected by their size, shape and surface characteristics 44. *In vivo* fluorescent imaging was employed to investigate the distribution of EDS NPs in the NSCLC xenograft mouse model. Two animal models were used in this experiment, and the tumors are indicated by circles and arrows in the figures. In Figure 5A, the mouse on the left was injected with free IR-780, and the mouse on the right side was treated with IR-780-loaded EDS NPs. Thirty minutes after injection, the signal in the mouse injected with free IR-780 was significantly lower than that in the EDS NP-treated mouse. However, in the EDS NP-treated mice, the fluorescence mainly accumulated in the thorax and abdomen within the first 1 h. After 2 h, the fluorescent signal in the tumor areas gradually increased, and the intensity exceeded that in the trunk after 6 h and peaked after 12 h. Although the fluorescence was also greatly concentrated in the thorax, the signal was significantly weaker there than in the tumor area. This phenomenon was similar to that reported by Yin, who found accumulation primarily in the liver and tumor that was attenuated in the liver 45. After 72 h, the fluorescence in the thorax and abdomen almost disappeared. The fluorescent distribution in tissues (Figure 5D) further demonstrated that EDS NPs could effectively be delivered and accumulate in tumors. As shown in Figure 5E, in the EDS NP-treated mice, the intensity of the signal in tumors was obviously higher than that in other organs. The results indicated that EDS NPs exhibited strong tumor-targeted effects. Moreover, reducing their accumulation in normal organs and tissues was the main cause of the decrease in side effects. The results of repetitive experiment were further demonstrated that EDS NPs has excellent tumorous targeting *in vivo* (Figure S2). The comparativeness and the repetitive experiment showed that the targeting of EDS NPs is effective and reliable, and the individual differences of mice were excluded.

### *In vivo* toxicity and antitumor effects

**Figure 6:**
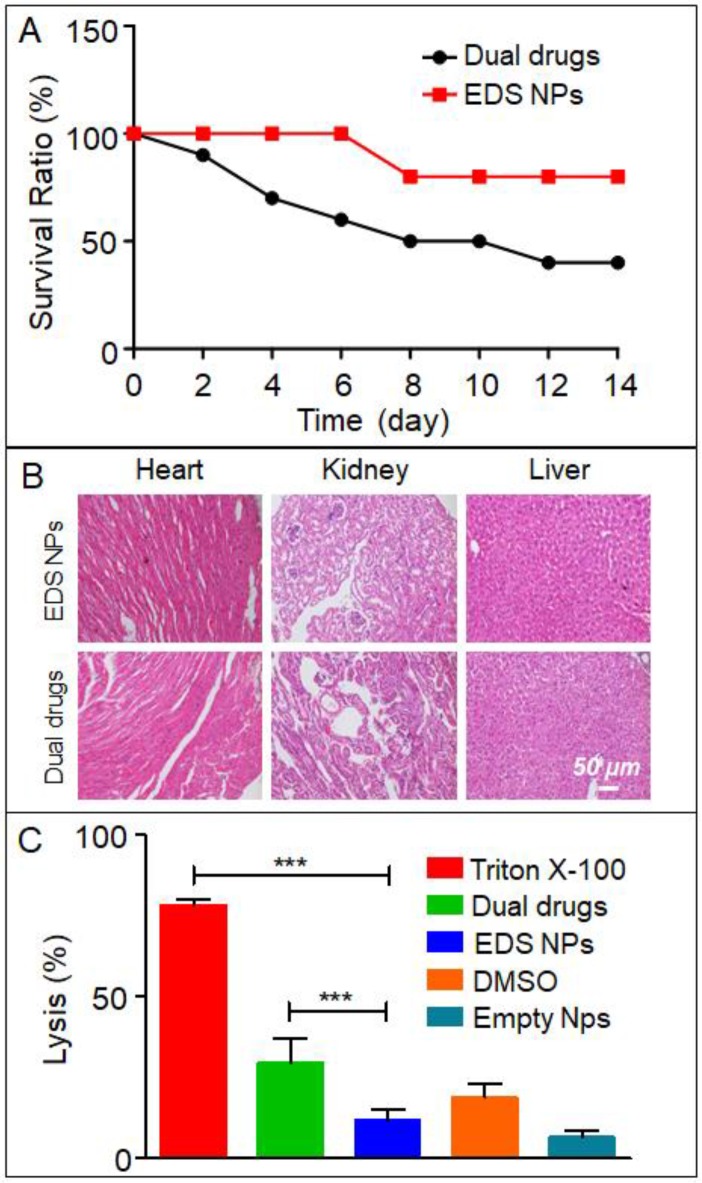
***In vivo* toxicity of EDS NPs.** Mice survival rate in acute toxicity (A). Pathological sections of heart, liver and kidney (B). Results of hemolytic test (C). Error bars represent the SD of the mean (n=3). The *** indicated p < 0.001 in t-test.

In the present study, an acute toxicity test was used to evaluate whether EDS NPs could decrease the *in vivo* toxicity of DOX and icotinib. The results are shown in Figure 6A. After injection with the same dose, the mortality in the EDS NPs-treated group was significantly lower than that in the dual drug combination-treated group. After 14 d, only 40% of the mice survived in the dual drug combination-treated group; in contrast, 80% of the mice in the EDS NP-treated group survived. The results of the examination of the pathological sections further demonstrated that the EDS NPs showed decreased toxicity in other organs. The results are shown in Figure 6B. In the dual drug combination-treated group, the cardiac tissues exhibited extravasated blood and inflammatory cell infiltration. Meanwhile, pathological changes were observed in the kidney, such as glomerulus atrophy, renal tubular necrosis and the dilation and congestion of small vessels in the mesenchyme. Moreover, injuries were detected in hepatic tissue. Hepatocytes exhibited irregular arrangements, swelling and the accumulation of lipid droplet vacuoles. In contrast, the tissues from the EDS NP-treated group only showed minor injuries. The results of the hemolysis assay are shown in Figure 6C. In the positive control group, Triton X-100 caused severe hemolysis, and the lysis rate was almost 80%. The DMSO-treated group exhibited more than 20% hemolysis. Treatment with the dual drug combination caused nearly 30% cell hemolysis. The hemolysis rate in the EDS NP-treated group was approximately 12%, and it was 7% in the empty NP group. The results indicated that EDC NPs could effectively decrease damage to erythrocytes caused by the drugs.

**Figure 7:**
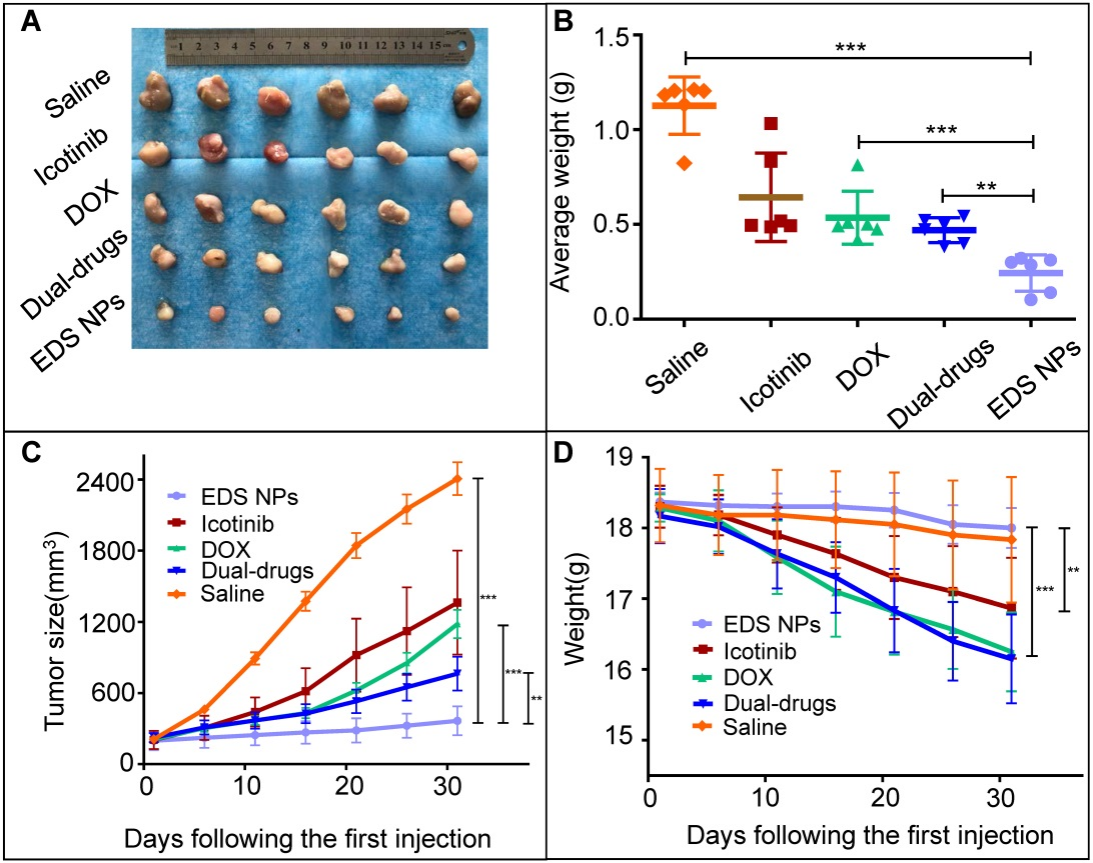
***In vivo* antitumor effect of EDS NPs.** The photo of tumor tissues (A). The weight of tumors (B). Tumor growth curves in treated groups (C). The changes of body weight in each groups (D). Error bars represent the SD of the mean (n=6). The * indicated p < 0.05 in t-test, ** indicated p < 0.01, *** indicated p < 0.001.

The *in vivo* antitumor evaluation was the last experiment in the present study. A549 cell xenograft mouse models were used for the experiment. The dosages of DOX and icotinib used were approximately 1 mg/kg and 10 mg/kg, respectively, according to the optimal proportion determined *in vitro*. The results showed that all treatment groups in the mice model exhibited tumor suppression (Figure 7). The single drug treatments inhibited tumor growth, and the average volumes were decreased approximately 6-fold and were approximately half of that found in tumors in the saline group. The tumors in the dual drug combination-treated group were smaller than those in the single drug treatment groups. Remarkably, the EDS NPs effectively suppressed the progression of NSCLC in the mouse models. The average volumes and weights of the tumors in the EDS NP-treated group were significantly smaller than those in the other groups.

**Figure 8:**
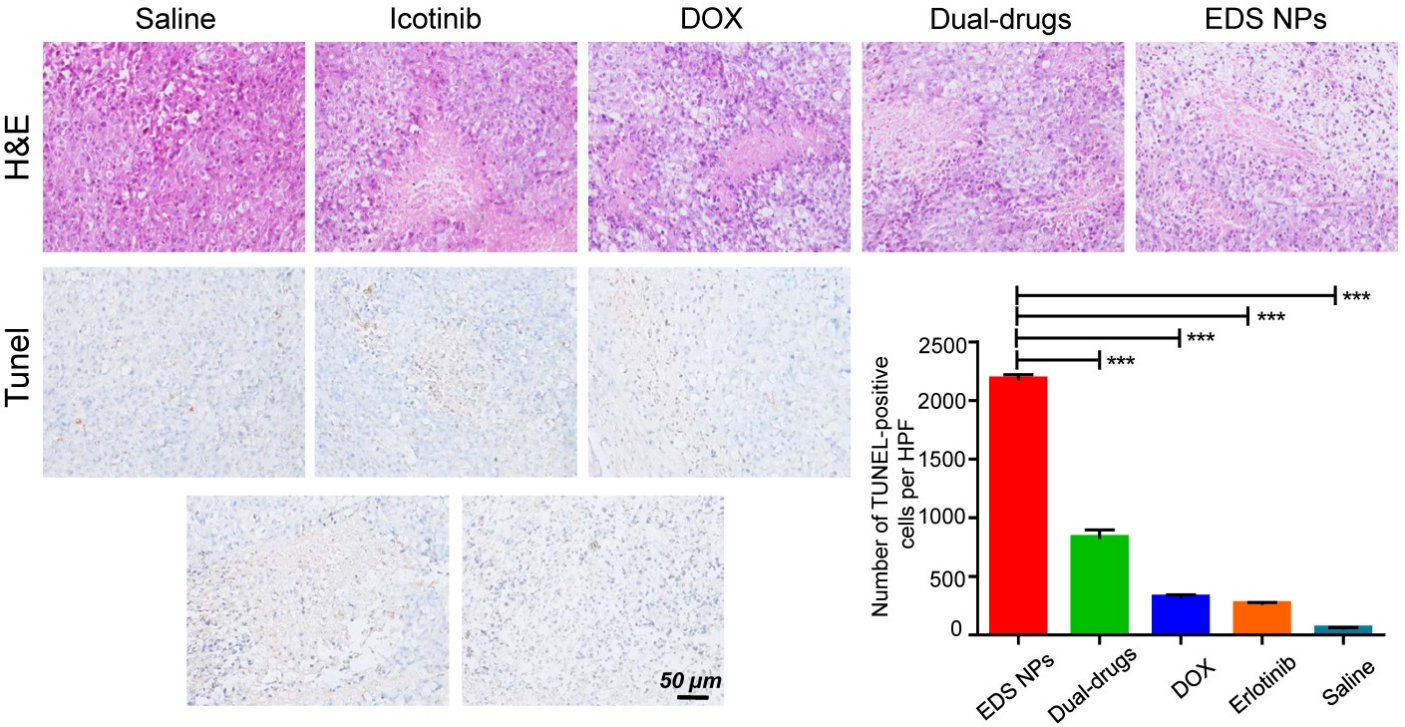
** Histological assay of tumors.** The photos of HE staining and TUNEL staining. Quantitative analysis of positive spots in TUNEL assay. Error bars represent the SD of the mean (n=6). The *** indicated p < 0.001 in t-test.

The histological investigation indicated that the tumors in the EDS NPs-treated group exhibited more pathological changes. In the sections used for TUNEL staining, the number of positive spots was signifycantly higher in the EDS NPs-treated group than in the other groups (Figure 8). The results indicated that EDS NPs could deliver more therapeutic agents into tumors. Moreover, the average body weight in the EDS NPs group was significantly higher than that in the other groups. This further demonstrated that *in vivo* antitumor treatment with the NPs was more effective. These results match those from the study by Lee, which demonstrated that EGFR inhibitors could effectively enhance apoptotic responses 9.

## Conclusions

In conclusion, the present study demonstrated that the use of EDS NPs was effective for *in vitro* and *in vivo* targeted delivery and the inhibition of NSCLC and greatly enhanced the synergistic effect of DOX and icotinib. Initially, icotinib was identified as the optimal synergistic counterpart for DOX. The drugs were coencapsulated by HA and CSaSt to construct the EDS NPs. The* in vitro* and *in vivo* results showed that EDS NPs could enhance the accumulation of drugs in tumor cells and reduce nonspecific accumulation in normal organs and tissues, thus effectively enhancing the curative effect and decreasing *in vivo* toxicity. This study suggests that functional NPs show noteworthy potential for the development of therapeutics for use in NSCLC clinical chemotherapy.

## Supplementary Material

Supplementary figures.

## Figures and Tables

**Scheme Schematic:**
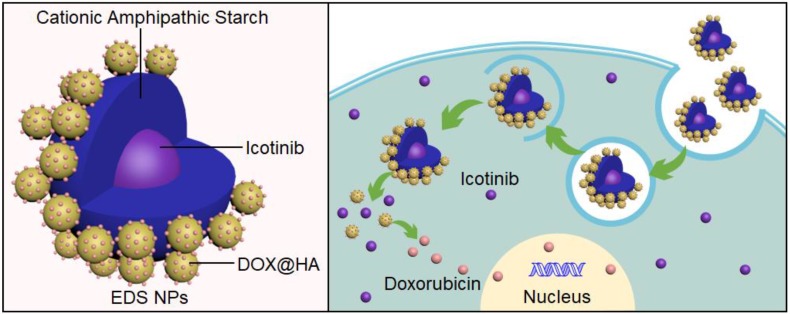
representation of structure and internalization of the EDS NPs.

**Table 1:** CI and dose reduction values for inhibition on A549, H1957 and PC9 by combining doxorubicin with icotinib

% Inhibition	CI	Doxorubicin				Icotinib		
		Conc.:(μmol/L)		Dose reduction		Conc.:(μmol/L)		Dose reduction
		Alone	Mix			Alone	Mix	
**A549**								
50	0.4178	0.784	0.134	5.851		16.947	1.273	13.312
75	0.4376	1.245	0.242	5.145		20.341	2.086	9.751
95	0.4788	1.879	0.398	4.721		34.854	3.172	10.988
**NCI-H1975**								
50	0.5892	0.872	0.347	2.513		20.436	2.807	7.280
75	0.5980	1.411	0.598	2.360		31.795	4.818	6.599
95	0.6863	1.942	1.002	1.938		38.167	8.053	4.739
**PC9**								
50	0.7322	0.214	0.118	1.814		1.321	0.956	1.382
75	0.7552	0.387	0.256	1.512		2.043	2.008	1.017
95	0.7887	0.512	0.313	1.636		2.919	2.536	1.151

**Table 2:** CI and dose reduction values for inhibition on A549, H1957 and PC9 by combining doxorubicin with erlotinib

% Inhibition	CI	Doxorubicin				Erlotinib		
		Conc.:(μmol/L)		Dose reduction		Conc.:(μmol/L)		Dose reduction
		Alone	Mix			Alone	Mix	
**A549**								
50	0.6527	0.784	0.357	2.196		17.432	3.021	5.770
75	0.7688	1.245	0.684	1.820		31.568	5.633	5.604
95	0.6743	1.879	0.887	2.118		46.173	7.099	6.504
**NCI-H1975**								
50	0.8012	0.872	0.554	1.574		14.355	4.848	2.961
75	0.7973	1.411	0.921	1.532		27.421	7.654	3.586
95	0.7743	1.942	1.143	1.699		41.465	8.563	4.842
**PC9**								
50	0.6894	0.214	0.093	2.301		0.943	0.711	1.326
75	0.6546	0.387	0.148	2.615		1.441	1.164	1.238
95	0.7102	0.512	0.279	1.835		1.986	1.537	1.292

**Table 3:** CI and dose reduction values for inhibition on A549, H1957 and PC9 by combining doxorubicin with apatinib

% Inhibition	CI	Doxorubicin				Apatinib		
		Conc.:(μmol/L)		Dose reduction		Conc.:(μmol/L)		Dose reduction
		Alone	Mix			Alone	Mix	
**A549**								
50	0.8545	0.784	0.523	1.499		28.998	4.445	6.523
75	1.0332	1.245	0.971	1.282		39.449	8.162	4.833
95	0.9853	1.879	1.435	1.309		54.518	12.751	4.276
**NCI-H1975**								
50	0.5675	0.872	0.231	3.775		13.193	1.242	10.622
75	0.6121	1.411	0.476	2.964		23.643	4.116	5.744
95	0.6798	1.942	0.712	2.728		39.532	5.532	7.146
**PC9**								
50	1.1964	0.214	0.201	1.209		24.682	1.888	13.073
75	1.0532	0.387	0.329	1.294		38.784	2.843	13.641
95	1.1723	0.512	0.483	1.270		51.343	4.032	12.733
