# Supplementary material for: Dual Loading of Nanoparticles with Doxorubicin and Icotinib for the Synergistic Suppression of Non-Small Cell Lung Cancer
Source: Int J Med Sci. 2020 Feb 4;17(3):390–402. doi: 10.7150/ijms.39172 (PMC7053357; doi:10.7150/ijms.39172)
Supplement: Supplementary file 1 — Supplementary figures. [file ijmsv17p0390s1.pdf]

Supplementary

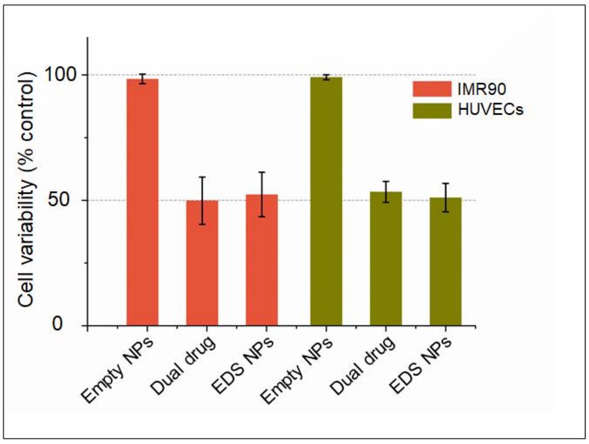

Figure S1: *In vitro* cytotoxicity of EDS NPs in two normal human cell lines, IMR90 and HUVECs. Error bars represent the SD of the mean (n=3).

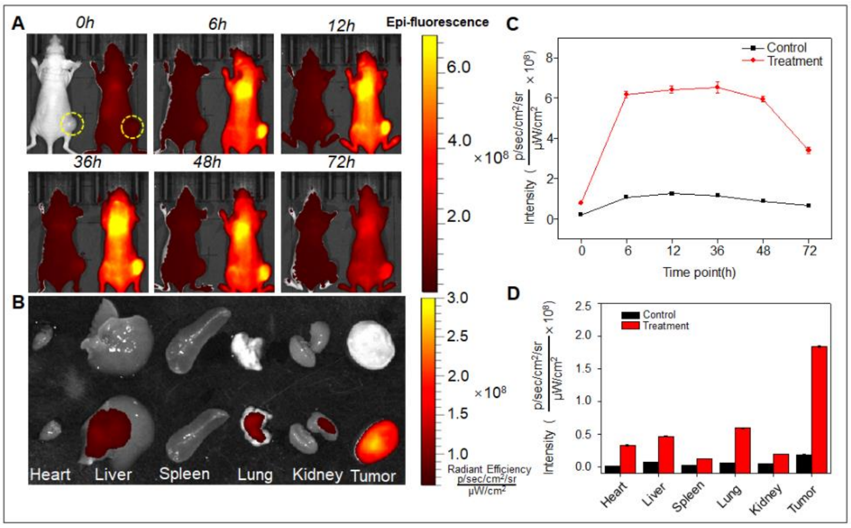

Figure S2: The repetitive experiment of *in vivo* targeted delivery of EDS NPs. The fluorescent signal distribution of EDS NPs during the experiment (A). Quantitative analysis of fluorescent intensity in mice (C). The fluorescent intensity in organs and tumors (B). Quantitative analysis of fluorescent intensity in tissues (D).
